# Circulating Tumor Cells in Glioblastoma

**DOI:** 10.3390/cancers18010010

**Published:** 2025-12-19

**Authors:** Robert H. Eibl, Markus Schneemann

**Affiliations:** 1 c/o M. Schneemann, Department of Internal Medicine, Hospitals of Schaffhausen, 8208 Schaffhausen, Switzerland; 2 Department of Internal Medicine, Hospitals of Schaffhausen, 8208 Schaffhausen, Switzerland; markus.schneemann@spitaeler-sh.ch

**Keywords:** circulating tumor cell, CTC, glioblastoma, GBM, liquid biopsy, clinical trials, biomarker, precision medicine

## Abstract

Glioblastoma multiforme (GBM) remains a devastating brain tumor with poor prognosis, traditionally viewed as non-metastatic. The recent detection of circulating tumor cells (CTCs) in glioblastoma challenges this long-held view and opens new opportunities for liquid biopsy in neuro-oncology. This review summarizes current understanding of glioblastoma CTCs, emphasizing their unique properties, detection technologies, and differences compared to CTCs in extracranial cancers. Key challenges include their rarity, the absence of epithelial markers, and the presence of the blood–brain barrier. Despite the need for specialized enrichment approaches, CTC analysis in glioblastoma can offer helpful information regarding tumor heterogeneity, treatment response, and minimal residual disease. We discuss emerging clinical studies leveraging CTCs for early relapse detection and therapy monitoring. Integrating CTC phenotyping with molecular and functional characterization may enhance future personalized treatment strategies in glioblastoma. Refined CTC methodologies combined with other liquid biopsy modalities may transform glioblastoma management, improving patient outcomes through less invasive, dynamic tumor surveillance.

## Simple Summary

Glioblastoma, the most aggressive primary brain tumor, has long been known to metastasize only in extremely rare cases, making systemic spread an exceptional finding. Nevertheless, recent advances have shown that glioblastoma cells can enter the bloodstream as circulating tumor cells (CTCs), challenging traditional assumptions about the disease’s strictly localized behavior. This review outlines current knowledge on glioblastoma CTC biology, detection methods, and clinical implications. Despite technical challenges—including their rarity and the lack of typical markers—glioblastoma CTCs provide a non-invasive way to monitor disease, guide treatment, and better understand tumor progression. Ongoing studies aim to improve CTC analysis sensitivity and integrate CTC insights with emerging therapies, ultimately advancing precision medicine for glioblastoma patients.

## 1. Introduction

Glioblastoma is the most common malignant brain tumor, with most patients surviving less than one year after diagnosis [1,2]. The detection of circulating tumor cells (CTCs) in glioblastoma patients was unexpected, as these tumors had long been considered non-metastatic (Table 1) [3,4,5,6,7,8]. This discovery, made little over a decade ago after about a century of glioblastoma research, contrasts markedly with observations in most extracranial cancers where tumor progression is typically accompanied by distant metastases and poorer survival. While CTC research has advanced in various cancers [9,10], important knowledge gaps and technical challenges remain in glioblastoma [11,12]. This review aims to clarify those gaps and challenges, focusing on CTC use in glioblastoma as compared to non-neural cancers and circulating tumor DNA (ctDNA) in liquid biopsy [13,14,15,16,17,18,19].

## 2. Circulating Tumor Cells (CTCs)

**Figure 1:**
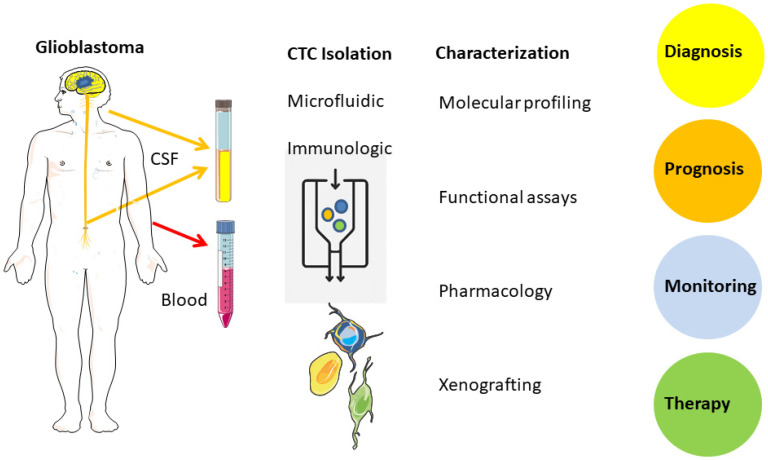
Circulating tumor cells (CTCs) in glioblastoma. Blood and cerebrospinal fluid (CSF) samples obtained distant from the primary brain tumor serve as minimally invasive and informative sources of tumor-derived cells. These samples can be used in downstream analyses aimed at predicting tumor progression and monitoring treatment response. Abbreviations: CSF, cerebrospinal fluid; CTC, circulating tumor cell. Figure created/modified with SMART and is licensed under the Creative Commons Attribution 3.0 Unported License. The Figure was adapted from Eibl and Schneemann, with permission [39].

Circulating tumor cells (CTCs) remain largely confined to experimental research compared to circulating tumor DNA (ctDNA), which has seen broader clinical adoption (Figure 1, Table 2) [11]. In contrast to ctDNA, CTCs preserve intact tumor cells, enabling phenotypic and functional analyses at the single-cell level. This review focuses on the differences between CTCs and ctDNA, and the challenges of applying CTC detection in glioblastoma, which include ultra-rare CTCs, lack of epithelial markers, and the protective blood–brain barrier.

## 3. Comparative Perspective on Liquid Biopsy Modalities in Glioblastoma

To place CTC-based analysis in context, it is useful to compare CTCs with other liquid biopsy approaches in glioblastoma, including ctDNA, extracellular vesicles (EVs), and CSF-based biomarkers. These modalities differ in biological material, analytic depth, sensitivity, and degree of clinical maturity. While ctDNA (particularly from CSF) is currently the most standardized approach for molecular monitoring in glioblastoma, CTCs uniquely preserve intact cells and therefore enable phenotypic and functional assessment at single-cell resolution. Table 3 summarizes the main strengths and limitations of each modality and highlights their complementary roles.

While ctDNA analysis has rapidly advanced toward clinical implementation, particularly in the context of cerebrospinal fluid sampling, its informative value remains largely restricted to genetic alterations. In contrast, CTCs provide intact cells that retain phenotypic, transcriptional, and functional characteristics. This distinction becomes relevant when studying treatment-induced cellular selection, phenotypic plasticity, or mechanisms of resistance that are not fully captured by cell-free nucleic acids. However, these potential advantages must be weighed against the markedly lower sensitivity of CTC detection compared with ctDNA assays. For this reason, CTC analysis is unlikely to replace ctDNA-based monitoring in glioblastoma. Instead, combined approaches integrating multiple analytes may offer a more comprehensive view of tumor biology, with ctDNA serving as a sensitive molecular readout and CTCs contributing complementary cellular information in selected research or translational settings.

## 4. Isolation, Enrichment, Characterization

CTCs can be isolated using microfluidic, immunological, and magnetic techniques [26,40]. CellSearch™ was the first FDA-approved platform (2004; Table 4) [25]. This platform isolates CTCs based on size, density, electrical properties, and immunophenotypic markers (EpCAM^+^, cytokeratin^+^, CD45^−^) and is cleared for use in breast, colorectal, and prostate cancer to aid in prognostic assessment. Glioblastoma CTCs are typically EpCAM-negative, so alternative methods like iChip, ScreenCell™, and pluriBead™ are under investigation (Table 4) [41].

The CTC-iChip combines multiple strategies to enrich CTCs based on physical properties like size and biological markers such as the epithelial cell adhesion molecule (EpCAM) [42]. While the positive selection for EpCAM is useful for most epithelial cancers, it is not suitable for glioblastoma because neural tumors usually lack EpCAM expression, but it has potential for glioblastoma, when the right markers will be applied; Specific CD44 variants may be an option [23]. Although GFAP is not a surface marker, it has been reported to support the identification of glioblastoma-derived tumor cells in other settings [43]. Methods like fluorescence in situ hybridization (FISH) have also been used to detect CTCs accurately in blood samples and can reveal chromosomal changes linked to different tumor types [44]. Recently, label-free microfluidic technologies have been developed which exploit the intrinsic physical characteristics of CTCs rather than relying on antibodies. These advances help lower costs and improve the feasibility of integrating CTC isolation into routine clinical workflows. Such innovations hold promise for broadening the clinical utility of CTCs, particularly in challenging contexts like glioblastoma. CTCs can be analyzed using DNA, RNA, and protein profiling. Whole exome sequencing (WES) and whole-genome sequencing (WGS) provide details of genetic heterogeneity and mutations at the single-cell level. Limited amounts of DNA from CTCs often require whole genome amplification (WGA) to check for single-nucleotide polymorphisms (SNPs) or copy number variations (CNVs).

## 5. Limitations and Current Barriers

Beyond biological constraints, technical and methodological factors further limit the current clinical applicability of CTC-based assays in glioblastoma. A major limitation is substantial inter-laboratory variability arising from differences in sample handling, enrichment technologies, marker selection, and analytical pipelines. Even when similar platforms are used, detection rates and reported CTC frequencies can vary substantially between centers, complicating cross-study comparisons and meta-analyses. In addition, the risk of false-positive CTC identification remains a relevant concern in brain tumor patients. Activated leukocytes, circulating endothelial cells, and therapy-induced inflammatory cell populations may partially overlap with tumor-associated phenotypes, increasing the risk of false-positive CTC identification. This issue is further exacerbated in glioblastoma by treatment-related blood–brain barrier disruption, which may transiently increase the release of non-malignant cells into the circulation. Finally, the ultra-rare nature of CTCs in glioblastoma introduces sampling bias and stochastic effects, especially when small blood volumes are analyzed. Together, these factors underscore the need for harmonized protocols, standardized quality controls, and multicenter validation efforts before CTC-based approaches can be reliably translated into routine clinical practice.

## 6. Glioblastoma vs. Extracranial Tumors

### 6.1. Extracranial Tumors

More than 150 years ago, Ashworth described morphologically identical cells in both blood vessels and in a skin metastasis—a finding that already reflected our current understanding of circulating tumor cells (CTCs) (Table 1) [20]. Two decades later, Paget proposed his seminal paper on the “seed and soil” theory, suggesting that metastatic cancer cells (“seeds”) can detach from their primary tumor and disseminate through the bloodstream to colonize distant sites with a favorable microenvironment (“soil”) [21]. This concept explained the unequal distribution of breast cancer metastases, which he found up to 15 times more likely to occur in the liver than in the spleen. Although only a small fraction of CTCs give rise to metastasis, their clinical detection is closely associated with tumor progression and metastatic spread [22]. Early detection of these ultra-rare—and hard to detect—CTCs can improve clinical decision-making, as well as monitoring tumor progression and treatment response. Only recently, the concept of CTCs, as well as their detection and characterization, entered clinical testing (Table 1). In 2004, Allard and colleagues introduced a robust workflow that enabled reliable detection and enumeration of CTCs from peripheral blood across several major carcinoma types, including prostate, breast, ovarian, colorectal, and lung cancer [25]. In the same year, Cristofanilli et al. reported that higher baseline CTC counts in metastatic breast cancer were independently associated with an unfavorable clinical course, reflected in shorter progression-free and overall survival [26]. Subsequent work extended CTC analysis beyond enumeration: in NSCLC, molecular characterization of DNA from isolated CTCs permitted longitudinal tracking of tumor evolution and EGFR mutational patterns under treatment, including changes linked to resistance [45]. The term “liquid biopsy” was introduced by Pantel and Alix-Panabières in 2010 to describe various methods for detecting and analyzing CTCs [40]. It has since been expanded to include circulating tumor DNA (ctDNA) and other tumor-derived materials from biofluids such as blood, cerebrospinal fluid (CSF), or urine. In 2013 Baccelli et al. identified a specific population of circulating tumor cells (CTCs) from breast cancer patients capable of initiating metastasis in a xenograft assay [9]. They isolated CTCs from breast cancer patients’ blood using specific surface markers including the stem cell marker CD44. These CTCs were then directly transplanted into the bone marrow of immunodeficient mice, which provided a niche similar to human metastatic sites. A subpopulation of CTCs expressing surface proteins CD44, CD47 (immune evasion signal “Don’t eat me”), and MET (a receptor that enhances migration/invasion) was able to initiate metastases in bones, lungs, and liver of mice. It showed that only a small fraction of heterogeneous CTCs with stem-like properties and a typical set of surface markers can initiate metastasis, due to better survival and colonization capabilities. These specific CTCs can serve as new targets to block the metastatic process. Liquid biopsy recently has been recognized as one of the major milestones in modern cancer research [46]. The gold standard of classical biopsy for solid tumors involves invasive procedures and provides a snapshot of the tumor. Minor subclones and metastatic lineages may not be detected, but are responsible for therapy resistance. Repeated surgical biopsies, especially from tumors in the brain, are less suitable for long-term monitoring. Comprehensive molecular profiling of primary tumor tissue is still less commonly applied. Depending on tumor location and patient comorbidities, repeated tissue sampling may be difficult or unsuitable for long-term monitoring. Moreover, a single tumor biopsy provides only a static snapshot that may fail to capture minor but progressive subclones, as well as molecularly distinct metastatic lineages responsible for therapy resistance. Therefore, liquid biopsy holds enormous potential as the next gold standard for monitoring tumor dynamics. Driven by advances in CTC detection and culture techniques, as well as a more profound understanding of tumor progression and metastasis, many clinical studies are currently underway to establish new monitoring standards for melanoma, breast, prostate, colorectal, and head and neck cancers. It was a major surprise recently, when glioblastoma, a highly malignant and devastating brain tumor known to almost never metastasize, was also found to regularly produce CTCs [3]. Early molecular and experimental studies have laid the conceptual foundation for current understanding of glioma biology and tumor cell migration. These include seminal work on TP53 alterations and molecular progression in astrocytic tumors, as well as oncogene-driven transformation and in vivo brain tumor models that enabled mechanistic studies of tumor growth, invasion, and dissemination under controlled experimental conditions [47]. Together, these studies continue to inform contemporary translational approaches, including liquid biopsy strategies and the interpretation of circulating tumor-derived material in glioblastoma.

### 6.2. Glioblastoma

**Figure 2:**
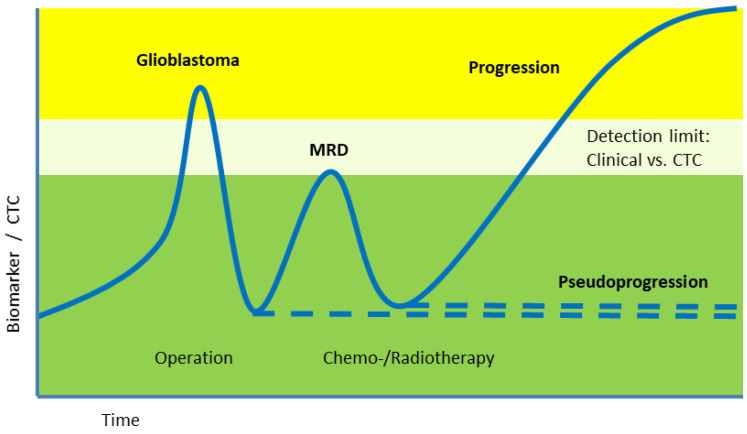
Hypothetical CTC dynamics during glioblastoma treatment. After tumor removal the CTC level should ideally drop. Detection of Minimal Residual Disease (MRD) by CTC may precede detection by standard clinical imaging methods. Figure created/modified with SMART and is licensed under the Creative Commons Attribution 3.0 Unported License. The Figure was adapted from Eibl and Schneemann, with permission [39].

In primary brain tumors, the blood–brain barrier represents an additional biological constraint that limits the passage of tumor cells into both the systemic circulation and the cerebrospinal fluid. At the same time, the current WHO classification increasingly emphasizes molecular profiling as a central diagnostic criterion for tumors of the nervous system, in some settings even outweighing conventional histopathological assessment [5]. Against this background, liquid biopsy approaches appear particularly attractive for longitudinal mutation monitoring in brain tumor patients, as they may reduce the need for repeated and potentially hazardous neurosurgical tissue sampling (Figure 2). In principle, ctDNA appears to be currently the most standardized source, but CTCs may also be used for specific mutational analysis [48,49]. CSF from brain tumor patients offers an additional source of information compared to blood. Therefore, a real-time liquid biopsy from CSF or blood offers a less invasive option to either monitor the tumor evolution, or to allow further functional assays on resistance mechanisms with living CTCs in cell culture and even xenograft models to evaluate treatment options.

### 6.3. Clinical Studies

NIH ClinicalTrials.gov lists only a limited number of studies investigating CTCs in glioblastoma (Table 5). These limited studies point out the importance of CTC analysis for the early detection of minimal residual disease (MRD)—even before clinical or radiographic evidence of recurrence. Such early detection could enable more timely therapeutic adjustments and potentially improve patient quality of life. The clinical potential of CTC measurements can be useful as new immunological treatment strategies evolve and may require, or at least incorporate, CTC monitoring as part of routine disease assessment.

## 7. Challenges

CTC-based liquid biopsy shows promise but requires higher sensitivity, glioblastoma-specific markers, and integration with functional assays. Combining CTCs with ctDNA may improve clinical monitoring, while novel technologies like QCM and AFM could advance functional characterization. The results may include specific markers, like glioblastoma-specific CD44 variants [23] (and Eibl, unpublished) since they don’t express the epithelial marker often used for many other cancers. Newly developing treatment options like immune based therapies may also benefit from evolving CTC detection strategies to monitor the disease and treatment outcome [53,54]. Some existing animal models for brain tumors should be checked for detectable CTCs [47,55]. Marker-based enrichment strategies represent a central bottleneck for glioblastoma CTC detection. Unlike epithelial cancers, where EpCAM provides a broadly applicable target, glioblastoma lacks a single, well-validated surface marker that combines sensitivity and specificity. Proposed alternatives such as CD44 or CD44 splice variants are biologically plausible but require further validation across larger patient cohorts and technical platforms. In addition, glioblastoma-associated markers may be shared with non-malignant neural or immune cell populations, increasing the complexity of downstream identification. These challenges highlight the need for multi-marker panels and integrated phenotypic approaches rather than reliance on single-marker selection strategies.

Several liquid biopsy studies report a measurable lead time for the detection of tumor recurrence compared with conventional clinical assessment or radiologic imaging. However, such comparisons should be interpreted with caution, as imaging technologies themselves continue to evolve. For example, the spatial resolution and detection sensitivity of modern high-field MRI systems (e.g., 3T or 7T) exceed those of earlier 1.5T platforms, introducing uncertainty when lead-time estimates are compared across different technological eras.

At present, only a limited number of studies and clinical trials have incorporated CTC analysis for treatment monitoring in cancer patients, and data for glioblastoma remain particularly sparse. Larger, multicenter studies will be required to define reproducible workflows, assess clinical utility, and determine whether CTC-based assays can be meaningfully integrated into future clinical guidelines. Beyond enumeration and molecular profiling, viable circulating tumor cells may enable exploratory functional and biophysical analyses that extend conventional liquid biopsy approaches. Atomic force microscopy (AFM)-based techniques have been widely used to characterize cancer cells by probing mechanical properties such as stiffness and viscoelasticity through indentation measurements, with early studies demonstrating clinically relevant differences in breast cancer cells compared to non-malignant counterparts [56]. More recently, technological advances have enabled high-throughput AFM platforms, including parallelized and automated measurement schemes, which substantially improved scalability and reproducibility with the intention to be adapted for the analysis of ultra-rare cell populations such as CTCs [57]. Complementary to mechanical phenotyping, AFM-based single-molecule force spectroscopy has been applied to study receptor–ligand interactions and signaling-dependent adhesion processes on living cells at molecular resolution [58,59,60]. Although these approaches remain experimental and have not yet been systematically applied to CTCs, they may offer long-term potential for hypothesis-driven functional characterization. In addition, label-free optical techniques such as Raman spectroscopy are increasingly explored for their ability to generate molecular fingerprints of individual cancer cells without the need for antibodies or prior labeling [61,62,63,64]. Although current implementations face challenges related to sensitivity and throughput, continued technical development may allow integration into multimodal CTC characterization workflows. Similarly, quartz crystal microbalance (QCM)-based approaches could conceptually enable dynamic monitoring of cell adhesion and drug-induced cytoskeletal responses in adherent tumor cells under controlled conditions [65]. At present, these technologies are best viewed as complementary experimental platforms rather than hyped candidates for routine clinical diagnostics, but they highlight the broader potential of functional assays in advancing CTC research. In this context, adherence to FAIR (Findable, Accessible, Interoperable, and Reusable) data principles may facilitate standardized sharing of phenotypic, functional, and molecular CTC datasets across studies, thereby supporting integrative analyses, reproducibility, and method development in liquid biopsy research [66].

To improve reproducibility, established models may test some of these experimental technologies first. The clinical validation of CellSearch-based CTC enumeration in breast cancer established CTCs as prognostically relevant biomarkers. Building on this principle, future liquid biopsy approaches may benefit from combining CTC analyses with ctDNA-based assays, rather than relying on a single analyte. Similar integrative strategies, such as the combination of mutation profiling with additional biomarker classes, have demonstrated improved performance in cancer detection and monitoring. From a translational perspective, the slow integration of CTC-based assays into neuro-oncology also reflects regulatory and logistical hurdles. Large, prospective multicenter studies are difficult to conduct in glioblastoma due to limited patient numbers, rapid disease progression, and heterogeneity in clinical management. Moreover, regulatory pathways for complex, multi-step cellular assays are less established than for sequencing-based diagnostics. Addressing these challenges will require coordinated efforts that combine methodological standardization with clinically meaningful endpoints. In this context, exploratory CTC studies should be designed to complement, rather than compete with, established liquid biopsy approaches, thereby maximizing their potential contribution to personalized glioblastoma management.

## 8. Conclusions

Circulating tumor cells provide biologically informative insights into glioblastoma biology but remain limited in their current clinical applicability. While CTC analyses have demonstrated prognostic and monitoring value in several systemic malignancies, their application in primary brain tumors remains constrained by low detection sensitivity, biological selection effects, and substantial technical variability. As a result, CTCs cannot presently be considered suitable for population-based screening or for reliable longitudinal monitoring in all patients with glioblastoma. In the clinical setting, liquid biopsy approaches based on circulating tumor DNA—particularly when derived from cerebrospinal fluid—currently offer a more robust and standardized framework for molecular disease assessment. In contrast, CTC analysis may add value in selected contexts by providing cellular and functional information that cannot be obtained from cell-free analytes alone. Such information may be relevant for exploratory assessment of treatment-induced tumor cell selection or resistance mechanisms, but its clinical impact remains to be defined. Further progress will require prospective clinical studies with harmonized methodologies to clarify where CTC-based assays can meaningfully complement established liquid biopsy strategies. Improvements in enrichment technologies, marker selection for EpCAM-negative tumor cells, and analytical standardization will be essential prerequisites. Until these challenges are addressed, CTCs in glioblastoma should be regarded as an investigational research tool rather than a routine clinical biomarker.

## 9. Future Directions

Future progress in CTC-based liquid biopsy for glioblastoma will depend less on conceptual novelty than on incremental but robust methodological advances. A primary priority is the systematic validation of marker panels suitable for EpCAM-negative glioblastoma CTCs, including mesenchymal and stem cell-associated markers, across independent cohorts and laboratories. Such efforts are essential to improve reproducibility and to enable broader implementation of marker-independent or hybrid enrichment platforms. In parallel, combined liquid biopsy strategies integrating CTC analysis with circulating tumor DNA (ctDNA) profiling are likely to offer complementary advantages. While ctDNA provides higher analytical sensitivity and is increasingly standardized, CTCs uniquely allow cellular, phenotypic, and functional characterization. Prospective clinical studies should therefore evaluate integrated workflows rather than competing modalities, particularly for monitoring minimal residual disease and treatment response. From a translational perspective, future studies should prioritize multi-omics characterization of glioblastoma CTCs at the single-cell level, combining genomic, transcriptomic, and proteomic data where feasible. Importantly, such approaches must be embedded in clinically realistic study designs, with clearly defined endpoints and harmonized protocols. Large, multicenter prospective trials will ultimately be required to determine whether CTC-based assays can move beyond exploratory research and contribute meaningfully to clinical decision-making in glioblastoma. Recent comprehensive reviews highlight the rapid technological evolution of circulating tumor cell research and underscore the need for clinically realistic study designs to facilitate translational progress in this field [41].

## Figures and Tables

**Table 1:** Circulating tumor cells (CTCs): Timeline of the original concept, experimental development, and progression toward clinical application. Parts of the table have been adapted from Eibl and Schneemann, with permission [11].

Year	Author(s)	Method	Tumor Type	Milestone
1869	Ashworth T.R. [20]	Autopsy; microscopy; case report	Unknown primary tumor	First description of tumor cells in blood; morphologically identical to metastatic lesions
1889	Paget S. [21]	Autopsy	Breast cancer	Formulation of the ‘seed and soil’ hypothesis of metastasis
1975	Fidler I.J. [22]	Experimental metastasis assay	B16 melanoma	Only a small fraction of injected tumor cells forms metastases
1995	Eibl R.H. et al. [23]	Molecular and functional characterization	Glioblastoma and astrocytoma	First detection of CD44 splice variants; potential CTC markers
2001	Reya T. et al. [24]	Stem-cell biology applied to cancer heterogeneity	Solid tumors and leukemia; migratory CSCs	Development of the cancer stem cell concept
2004	Allard W.J. et al. [25]	CellSearch™	Prostate, breast, ovarian, CRC, lung cancers	CTC detection in 7.5 mL blood samples
2004	Cristofanilli M. et al. [26]	CellSearch™ (CTC enumeration)	Metastatic breast cancer	CTCs as independent predictor of reduced PFS and OS
2008	Maheswaran S. et al. [27]	Molecular profiling; EGFR mutation detection	NSCLC	CTC-based therapy monitoring
2008	Cohen S.J. et al. [28]	CellSearch™; clinical study	Colorectal cancer	Clinical feasibility of CTC enumeration
2008	De Bono J.S. et al. [29]	Clinical study	Prostate cancer	CTC count as strongest independent predictor of OS
2010	Pantel K., Alix-Panabières C. [30]	Conceptual review	Metastatic cancers	Introduction of the term ‘liquid biopsy’
2013	Dawson S.J. et al. [31]	Disease monitoring	Breast cancer	ctDNA more sensitive than CTCs for therapy monitoring
2013	Baccelli et al. [9]	Xenograft	Breast cancer	Identification of metastasis-initiating CTC subsets
2014	Sullivan J.P. et al. [3]	CTC-iChip (negative depletion)	GBM	Demonstration of CTCs in glioblastoma
2014	Neves R.P. et al. [32]	Microfluidic enrichment	NSCLC	EGFR variant detection via single-cell sequencing
2014	Polzer B. et al. [33]	CTC genome/transcriptome profiling	Breast cancer	Diagnostic potential; heterogeneity to primary tumors
2015	Mazel M. et al. [34]	CellSearch™	Breast cancer	PD-L1 detection on CTCs
2018	Krol et al. [35]	—	GBM	Identification of CTC clusters in blood
2019	Szczerba P. et al. [36]	CTC analysis	GBM	Neutrophils escort CTCs; support proliferation and metastasis
2019	Gkountela S. et al. [37]	CTC analysis	GBM	CTC clusters show distinct methylation and higher metastatic potential
2023	Chowdhury et al. [38]	Advanced CTC detection; single-cell profiling	Various cancers	Technical advances in CTC analysis

**Table 2:** Clinical utility of CTCs.

Pro	Con	Clinical Utility
Sufficient sensitivity in some advanced cancers	Limited sensitivity in screening, early-stage cancers, many advanced cancers	Prognostic markers in metastatic breast, prostate, colorectal cancers
FDA-approved enumeration for specific applications	Not standardized, experimental methods; centralized high-tech laboratories	Prediction of relapse, incl. treatment response using living CTCs (cell culture, xenograft)
High specificity (mutations)	Sophisticated technology, no easy/common standards; expensive; no remuneration; extra challenges for brain tumors (lacking epithelial markers)	Clinical potential; research use; high cost; limited availability

**Table 3:** Comparison of liquid biopsy approaches in glioblastoma (GBM).

Liquid Biopsy	Material	Strengths	Limitations	Clinical Maturity
Circulating tumor cells (CTCs)	Intact, viable tumor cells	Preserve cellular phenotype; allow functional assays; enable single-cell multi-omics; potential insight into invasion and resistance	Ultra-rare; no standardized markers; EpCAM-negative phenotype in GBM; technical variability; limited validation	Exploratory/research use
Circulating tumor DNA (ctDNA)	Fragmented tumor-derived DNA	High specificity for mutations; increasingly standardized assays; suitable for longitudinal monitoring; CSF often informative	No cellular/functional information; limited sensitivity in plasma for CNS tumors; reflects mainly genomic alterations	Closest to clinical routine
Extracellular vesicles (EVs)	Vesicles carrying proteins, RNA, DNA	Relatively stable; reflect active secretion; multi-analyte potential	Heterogeneous populations; tumor attribution can be difficult; limited standardization	Experimental/early translational
CSF biomarkers (proteins, ctDNA)	Cell-free molecules in CSF	Higher proximity to CNS tumors; improved sensitivity vs. blood in many settings	Invasive sampling; not suitable for frequent monitoring in all patients	Translational/selective clinical use

**Table 4:** CTC isolation methods and applicability to glioblastoma (GBM).

Method	Principle	Characteristics	Utility for GBM
CellSearch™	EpCAM-based immunomagnetic selection	FDA-cleared; isolates EpCAM-positive CTCs; cytokeratin/CD45 staining; automated workflow	Not suitable (GBM typically EpCAM-negative)
iChip	Microfluidic inertial focusing + immunomagnetic depletion	High-throughput, marker-independent; preserves viability and heterogeneity	Research tool; potential with GBM-specific markers
ScreenCell™	Microfiltration based on cell size (antigen-independent)	Fast, antigen-independent; efficient for heterogeneous viable CTCs	Suitable for EpCAM-negative GBM
pluriBead™	Bead sieving with bound target cells	High purity, gentle isolation; minimal blood contamination	Potentially advantageous for rare GBM CTCs

**Table 5:** Selected clinical studies using CTCs in glioblastoma (GBM).

Year	Study	Tumor	Outcome Measure
2016	Gao et al. [44]	GBM, other gliomas	CTC incidence
2018	Liu et al. [50]	GBM	Similarity of GBM CTCs with CSC (in both mice and humans)
2019-21	NCT03861598 [51] Early phase 1 study (Morgantown, WV, USA)	GBM	Carvedilol added to standard chemotherapy, correlating MRI controls with new RT-PCR test for CTC detection
2021	Müller-Bark et al. [48]	GBM	CTC number post-surgery correlated with survival
2021-25	GLIOLIPSY: LIQUID BIOPSY IN Low-grade Glioma Patients NCT05133154 [52] Interventional study (University Hospital, Montpellier)	Low-/High-grade glioma	Pre- and post-surgery detection and characterization of CTC and TEP
2023-27	INCIPIENT: INtraventricular CARv3-TEAM-E T Cells for PatIENTs With GBM NCT05660369 Phase 1 study (MGH, Boston, MA, USA)	GBM	CAR-T cell study (dose/safety) in glioblastoma with EGFRvIII mutation, incl. CTC analysis

CSC—Cancer stem cell; NCT numbers refer to NIH ClinicalTrials.gov study numbers; TEP—Tumor-Educated Platelets.

## Data Availability

No new data were created or analyzed in this study. Data sharing is not applicable to this article.
